# Patients With Dementia Undergoing High-Risk Inpatient Surgery Have Poor Outcomes

**DOI:** 10.1111/jgs.70091

**Published:** 2025-09-16

**Authors:** Samir K. Shah, Lingwei Xiang, Rachel R. Adler, Clancy J. Clark, Zara Cooper, Emily Finlayson, Susan L. Mitchell, Dae Hyun Kim, Kueiyu Joshua Lin, Stuart R. Lipsitz, Joel S. Weissman

**Affiliations:** 1 Division of Vascular Surgery, University of Florida, Gainesville, Florida, USA; 2 Center for Surgery and Public Health, Brigham and Women’s Hospital, Boston, Massachusetts, USA; 3 Division of Surgical Oncology, Wake Forest School of Medicine, Winston-Salem, North Carolina, USA; 4 Department of Surgery, Phillip R. Lee Institute for Health Policy Studies, University of California, San Francisco, California, USA; 5 Hebrew SeniorLife, Arthur and Hinda Marcus Institute for Aging Research, Boston, Massachusetts, USA; 6 Division of Pharmacoepidemiology and Pharmacoeconomics, Department of Medicine, Brigham and Women’s Hospital, Boston, Massachusetts, USA

**Keywords:** dementia, high-risk surgery, Medicare

## Abstract

**Background:**

Surgery is common among patients living with dementia, and understanding outcomes may help decision-making. We compared patients’ and utilization outcomes after high-risk surgery among patients with and without dementia.

**Methods:**

In this retrospective national cohort study, we compared outcomes of Medicare fee-for-service beneficiaries 66 years or older who underwent high-risk inpatient surgery (i.e., with an inpatient mortality of at least 1%) from January 1, 2017 to September 30, 2018. We examined 90- and 30-day all-cause mortality, major complications, discharge to a higher level of care, intensive end-of-life interventions, and prolonged skilled nursing facility (SNF) stay. We used generalized estimating equations regression modeling and competing risks analysis.

**Results:**

Among 19,998,187 beneficiaries, we identified 859,570 who had fee-for-service coverage and were 66 years or older at the time of a high-risk surgery. Of these, 124,822 (14.5%) had a diagnosis of dementia. Female sex accounted for 81,252 (65.1%) of the dementia cohort. Four of five of the most common procedures were related to femur fracture and cardiac surgery in the dementia and non-dementia cohorts. Ninety-day mortality was worse among patients with dementia: 22.8% versus 9.3% (adjusted odds ratio [aOR] 1.82, 95% confidence interval [CI] 1.78–1.85). Patients with dementia were also more likely to have major complications (51.6% vs. 38.5%, aOR 1.19, 95% CI 1.17–1.20), be discharged to a higher level of care (75.1% vs. 41.3%, aOR 1.49, 95% CI 1.44–1.53), and have a prolonged SNF stay (3.7% vs. 1.4%, aOR 1.80, 95% CI 1.69–1.91). Although patients with ADRD were overall less likely to receive intensive interventions during the index admission and at 90 days, they were more likely to receive feeding tubes (aOR 1.22, 95% CI 1.17–1.28).

**Conclusion:**

Persons living with dementia experience a broad range of worse outcomes after high-risk surgery compared to those without dementia. These data may be used for decision-making.

## 1 | Introduction

Inpatient surgery among older Americans is common—over 2 million inpatient surgeries are performed annually in the US on patients 65 years or older [1]. Among these, persons living with Alzheimer’s disease and related dementias (ADRD) represent a group at particular risk for mortality, readmissions, and other adverse events during and after surgical admission [2, 3]. Goal-concordant medical care is a mark of high-quality care but itself rests on the foundation of outcomes data. Existing outcomes data on ADRD patients undergoing surgery tend to include only limited institutional data [2] or patients residing in nursing homes [4, 5]. Furthermore, much of the surgical literature focuses on particular conditions and surgeries and is limited in critical ways, such as lacking longer-term post-discharge outcomes [6–9]. While procedure-specific data are ideal, such data are not currently available for most surgeries. These gaps in knowledge complicate decision-making for patients and their families. For example, there are no outcomes data specific to mesenteric bypass, an invasive and complex procedure, in patients living with ADRD. Patients and their families facing this surgery are left to the surgeon’s judgment and extrapolation from outcomes data in patients without ADRD for decision-making. In these cases, an understanding of outcomes after high-risk surgery can provide important insights into hospital length of stay, mortality, risk of discharge to higher levels of care, and other outcomes meaningful to patients and families. Similarly, these data may also help inform advance care planning for patients not imminently facing surgery. Although commonly used, “major surgery” has no consensus definition, and so we have focused instead on “high-risk surgery,” which we define as having an inpatient mortality of at least 1% [10]. In this study, we sought to measure both traditional (e.g., 90-day mortality) and patient-centered (e.g., risk of discharge to a higher level of care) outcomes for patients with and without ADRD undergoing high-risk inpatient surgery.

## 2 | Methods

This study was approved by the Mass General Brigham institutional review board. Waiver of informed consent was obtained.

### 2.1 | Data Sources and Study Population

We used fee-for-service Medicare claims data from January 1, 2016, to December 31, 2018, and examined Medicare beneficiaries 66 years or older who underwent high-risk inpatient surgery between January 1, 2017, and September 30, 2018. We excluded all patients without continuous fee-for-service enrollment in the preceding 12 months and the 3 months after surgery. We used a previously published list of high-risk inpatient surgery [1]. In brief, to generate this list of high-risk surgeries, we identified all procedures with a crude inpatient mortality of at least 1% irrespective of procedure urgency—in line with the definition of high-risk by Schwarze et al.—among 2,241,249 fee-for-service beneficiaries in 2018 Medicare data [11]. Three board-certified surgeons at different institutions independently reviewed the list to exclude procedures with high mortality that likely resulted because they occurred in high-risk medically complex patients rather than intrinsic risk (e.g., tracheostomy creation). To validate our list, we examined the mortality of each code among fee-for-service beneficiaries in 2019–2020 and found that 224 of the original 231 (97.0%) of codes retained an inpatient mortality of 1% or higher [1]. For this study, we also excluded percutaneous procedures (e.g., percutaneous aortic valve replacement).

ADRD was defined dichotomously based on ICD-10 diagnosis codes during a 12-month look-back period or index admission. Our codes identifying ADRD are based on chart validation and expert consensus, exclude codes for nonspecific and reversible conditions, and have a positive predictive value of 77.1% [12].

### 2.2 | Covariates and Outcomes

We collected demographic information from the Medicare Beneficiary Summary Files. Age was calculated by using the beneficiary’s birth date and the date of index surgery. Research Triangle Institute race code was utilized in our analysis [13]. Data on comorbid conditions were collected by examining all ambulatory and inpatient files during the 12-month look-back period and was summarized using the van Walraven modification of the Elixhauser comorbidity index [14]. Patients were dichotomized into frail versus non-frail patients using a claims-based frailty index (cFI) and a cutoff of ≥ 0.250 [15]. The cFI is calculated using 93 variables derived from ICD diagnosis, Current Procedural Terminology-4 (CPT-4), and Healthcare Common Procedure Coding System (HCPCS) codes [15]. It has been validated against clinical frailty measures and outcomes including mortality, mobility impairment, and disability [15, 16]. Admission urgency was classified as urgent/emergent or elective if the admission source was the emergency room or the admission type was listed as “emergency” or “urgent.”

We first examined mortality 90 days after surgery. Next, we examined mortality 30 days after surgery and major inpatient complications following the surgical procedure. Major complications were defined as generic postoperative complications (e.g., myocardial infarction) and were guided by prior reports [2, 17, 18]. There is no consensus on the definition of a “major complication” in the surgical literature, and our list is a result of a twofold process consisting of examining existing published work such as by Dimick et al., Sheetz et al., and the American College of Surgeons’ National Surgical Quality Improvement Program, combined with review by surgeon scientists (including authors SS and CC) [19–21]. Discharge to a higher level of care was examined for all beneficiaries except those coming from a long-term acute care facility (LTAC) using the following hierarchy of discharge destinations: home<subacute or intermediate care skilled nursing facility (SNF) < LTAC. Discharge to home was calculated only for patients admitted from home. For patients admitted from home, the time-at-home ratio was defined as the proportion of time spent at home to time spent elsewhere from the date of surgery to 90 days after surgery or the date of death or end of the study period, that is, through December 31, 2018. SNF stays of 100 days or more were defined as prolonged. Length of stay was calculated from the date of the high-risk surgery to the date of discharge or in-hospital death. We examined intensive interventions during the index admission and within 90 days of surgery. Our list of intensive interventions was compiled using literature review and multi-disciplinary expert consensus and consists of cardiopulmonary resuscitation (CPR), tracheostomy, extracorporeal membrane oxygenation (ECMO), new renal replacement therapy (RRT), prolonged intubation, and surgical feeding tube placement (i.e., tubes such as percutaneous endoscopic gastrostomy in contradistinction to transnasal tubes). Except for CPR, ECMO, and intubation, these interventions were included only when they were not part of the index procedure nor part of a procedure within 1 year prior to index admission. Prolonged intubation was defined as mechanical ventilation greater than 96 h after surgery.

### 2.3 | Statistical Analysis

Continuous data were described using means and medians. Categorical data were described using frequencies and percentages. Unadjusted differences in outcomes between patients with and without ADRD were determined using Wilcoxon rank-sum tests and chi-squared tests or Fisher’s exact test as appropriate. We used generalized estimating equations (GEE) to account for clustering within the hospital. For dichotomous and continuous outcomes, GEE logistic regression and GEE linear regression were applied, respectively. In addition to GEE, we calculated inverse-propensity weighting (IPW) weights and conducted survival analysis using the Cox proportional hazard model and Fine-Gray competing risks analysis to account for death as a competing risk for intensive interventions on the index admission and within 90 days of surgery, prolonged SNF stay, and hospital length of stay. Models were adjusted for patient-level (age, sex, race, Elixhauser index score, frailty, dual Medicare/Medicaid eligibility) and procedure-level (admission urgency, procedure risks, number of high-risk surgeries performed during the index admission) factors. Unadjusted inpatient mortality from our prior data used to derive lists of high-risk surgery was used as a measure of procedural risk in regression models. The same covariates were included in the logistic model for ADRD to obtain IPW weights for each patient. All analyses were performed using SAS 9.4 (SAS, Cary, NC).

## 3 | Results

### 3.1 | Patient Characteristics

Among 19,998,187 Medicare beneficiaries from 2017 to 2018, we identified 859,570 beneficiaries age 66 years or older at the time of an inpatient high-risk surgery with continuous fee-for-service coverage (Figure S1). Among 859,570 individuals in our study, 124,822 (14.5%) had a diagnosis of ADRD (Table 1). Female sex accounted for 466,630 (54.3%) of the entire cohort and 81,252 (65.1%) of the ADRD cohort. The median age of the ADRD cohort was significantly higher than the non-ADRD cohort: 84.6 versus 75.7 years, *p* < 0.0001. The racial composition of the two cohorts was different (*p* < 0.0001) with a lower proportion of non-Hispanic Whites (83.7% vs. 86.2%) and higher proportions of non-Hispanic Blacks (8.1% vs. 5.8%) and Hispanics (5.1% vs. 4.1%) in the ADRD cohort. The mean Elixhauser index score was significantly higher in the ADRD cohort (15.8 vs. 13.2, *p* < 0.0001). Uncomplicated diabetes, hypertension, renal failure, and congestive heart failure were all more common in patients with ADRD. The ADRD cohort also had more patients with frailty (43.3% vs. 18.7%, *p* < 0.0001) and dual Medicare/Medicaid eligibility (33.6% vs. 13.8%, *p* < 0.0001).

Admission sources differed between the two groups. However, a large majority of both ADRD and non-ADRD patients were admitted from home, 82.0% and 89.8%, respectively (Table 1). Higher proportions of the ADRD cohort were transferred from another hospital (8.7% vs. 8.3%, *p*-value < 0.0001) or another facility, such as intermediate or subacute SNF (9.0% vs. 1.8%, *p*-value <0.0001).

### 3.2 | Procedural Characteristics

Of all patients undergoing high-risk procedures, 46.6% (400,140) underwent procedures that were classified as urgent or emergent, while the remainder were classified as elective (Table 2). There were more patients with ADRD undergoing urgent/emergent procedures than those without ADRD: 72.7% vs. 42.1%, *p* < 0.0001. Mean procedure risk (representing unadjusted mortality from our work on high-risk surgery, see Section 2) was lower in patients with ADRD (3.6% vs. 4.4%, *p* < 0.0001). The five most common high-risk procedures for each group are listed in Table 3—four out of five in the ADRD group are related to femur fracture, while four out of five for non-ADRD patients are cardiac surgeries. A full list of surgeries is provided in Table S1.

### 3.3 | Outcomes

**FIGURE 1 |:**
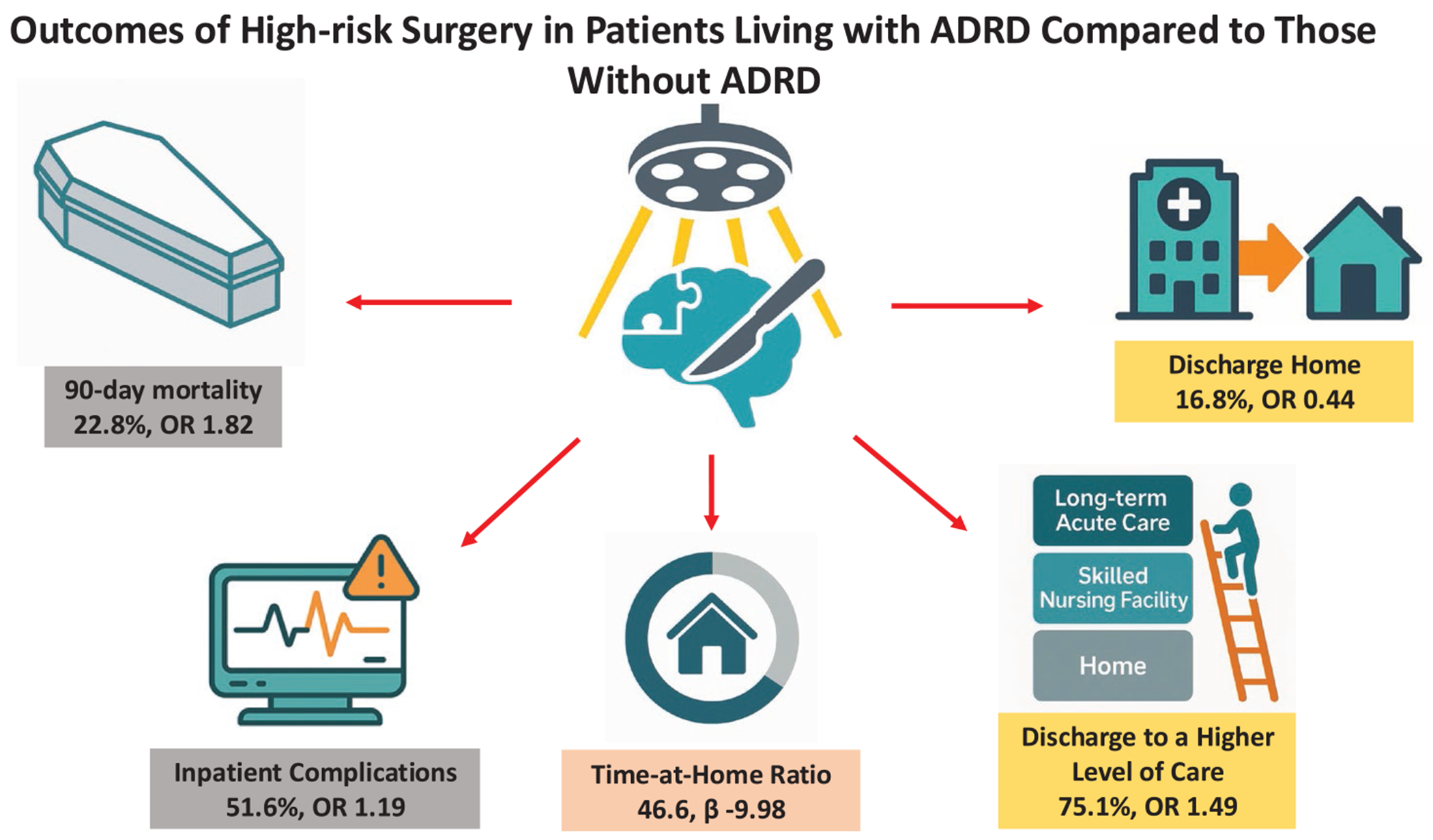
Outcomes of high-risk surgery in patients living with ADRD compared to those without.

Nearly all outcomes in patients living with ADRD were worse compared to those without ADRD (Figure 1). Mortality in the group with ADRD at 30 days was 12.6% versus 5.7% among those without ADRD. Mortality at 90 days showed a starker difference: 22.8% versus 9.3%. After adjustment, patients with ADRD were at higher risk for mortality at both 30 and 90 days, aOR 1.58 (95% CI 1.54–1.62) and 1.82 (95% CI 1.78–1.85). Just over half of patients with ADRD experienced a major complication compared to 38.5% of patients without ADRD (Table 4; Table S2 for a full list). After adjustment, patients with ADRD were more likely to have major complications than those without ADRD (aOR 1.19, 95% CI 1.17–1.20). Patients with ADRD experienced a 5.9 day mean hospital length of stay compared to 6.1 days in the non-ADRD group (adjusted mean difference −0.22, 95% CI −0.27 to −0.17).

Intensive interventions as a group were less common among those with ADRD during the index admission (aOR 0.85, 95% CI 0.82–0.88) compared to patients in the non-ADRD cohort. Each component intervention was also less common except for placement of a feeding tube (aOR 1.22, 95% CI 1.17–1.28). At 90 days there were no differences in the likelihood of intensive interventions overall between the two groups, but individual interventions were less likely in the ADRD group except for feeding tube placement, which was more likely: aOR 1.41, 95% CI 1.35–1.47.

Discharge and post-discharge outcomes were worse in the ADRD cohort. Using our prespecified hierarchy of discharge destinations (home<subacute or intermediate care SNF < LTAC), three out of four patients in the ADRD group were discharged to a higher level of care compared to 41.3% in the non-ADRD cohort, aOR 1.49, 95% CI 1.44–1.53. Among patients admitted from home, discharge to home occurred in 16.8% of patients with ADRD compared to 56.2% of patients without ADRD. The adjusted OR of discharge to home for persons living with ADRD was 0.44, 95% CI 0.43–0.50. Patients with ADRD were more likely to experience prolonged SNF stays, aOR 1.80, 95% CI 1.69–1.91. Overall, the mean time-at-home ratio was 46.6 for patients in the ADRD cohort compared to 73.0 for those in the non-ADRD cohort. This remained significant after adjustment (adjusted mean difference −9.98, 95% CI −10.25 to −9.71).

We repeated our analysis modeling death as a competing risk to reexamine hospital length of stay, prolonged SNF stay, and intensive interventions during the index admission and at 90 days. In line with our findings using GEE multivariable logistic regression, patients with ADRD were more likely to have longer SNF stays (discharge HR 0.94, 95% CI 0.93–0.95) and less likely to have intensive interventions during the index admission (HR 0.92, 95% CI 0.88–0.96) than those without ADRD when using competing risks analysis. In contrast, competing risks analysis gave opposing results and suggested a longer hospital length of stay (discharge HR 0.94, 95% CI 0.93–0.95) and higher likelihood of intensive interventions at 90 days (HR 1.13, 95% CI 1.08–1.18) for those with ADRD (Table S3).

## 4 | Discussion

Our data demonstrate that patients living with ADRD experience poor outcomes when undergoing high-risk inpatient surgery in an array of patient-centered and traditional measures ranging from 30- to 90-day mortality to the likelihood of prolonged SNF stay and home discharge. Further, outcomes for those living with ADRD are in every case worse than those without ADRD except for the risk of intensive interventions during the index admission.

These data may be used to help inform goal-concordant care. We have used “decision-making” and “goal-concordant care” interchangeably in this study and use both to refer to the provision of care in line with the intentions of the patient as outlined in an initiative of the National Academy of Medicine [22]. There are two scenarios in which our data could be used to this end: (1) advance care planning without any impending surgery and (2) decision-making around surgery in the absence of procedure-specific data. As an example of the former, consider a patient for whom living at home was a priority. They could use our data on likelihood of discharge to a higher level of care, prolonged SNF stay, hospital lengths of stay, and time-at-home ratio to understand that undergoing a high-risk surgery would likely be inconsistent with that goal. With regard to patients facing a high-risk surgery for which there is no procedure-specific data, we recognize that every procedure has specific nuances (e.g., unique complications, recovery trajectories, etc.) that our data do not capture. Nevertheless, they can serve as a first approximation of outcomes to begin the conversation around treatment options.

This study is novel because of the breadth of procedures examined and use of national data. Many prior studies have focused on single procedures, such as hip fracture or cancer surgery [7, 9, 23], but few have looked at larger sets of procedures. While procedure- or condition-specific studies may be immensely valuable for cases that are specifically relevant (e.g., outcomes of hip fracture for a patient with a hip fracture), studies of broader sets of surgery are better suited to more generalized decision-making as occurs, for example, in the setting of advance care planning for patients who are thinking through health care goals and values apart from a specific planned surgery. Additionally, our analysis explicitly focuses on risk to categorize surgeries rather than anesthetic approach or other features potentially unrelated to risk. For example, some authors have defined “major surgery” as a procedure “requiring the use of general anesthesia for a nonpercutaneous nonendoscopic invasive operation” [24]. The latter includes a broad spectrum of cases from low-risk (e.g., toe amputation) to high-risk (e.g., open aortic valve replacement) and would not be as useful for decision-making. Even within the category of “high-risk,” many have considered inpatient mortality without any consideration of the fact that some procedures, such as tracheostomy, are not intrinsically high-risk but tend to occur in patients at high risk for mortality [25]. Others have created limited lists of common high-risk surgeries without any attempts to be comprehensive resulting in the exclusion of important procedures such as neurosurgical procedures and common vascular procedures like thoracic aortic aneurysm repair [26]. There are numerous other approaches including the use of postsurgical admission to an ICU to define high-risk and a focus primarily on patient rather than procedural risk [27–29]. Our approach, while limited by a focus on procedure-derived risk rather than patient-derived risk, overcomes these shortcomings.

Existing studies that examined more than a single procedure or condition broadly support our findings. In particular, two studies reinforce our data demonstrating higher frequencies of inpatient complications and mortality [30, 31]. These two studies, however, did not examine other endpoints that we considered important, such as the risk of discharge to a higher level of care. Masutani et al. examined the Premier Healthcare Database for the 10 most common procedures in patients older than 65 and found a higher inpatient mortality, longer length of stay, and higher risk of discharge to a higher level of care for patients living with ADRD [8]. Importantly, however, this study assessed only inpatient outcomes. Bekelis et al. also reported findings broadly similar to those of Masutani but analyzed only nine high-risk surgeries [32]. These did not include hip surgery, one of the most common procedures, and were based on New York state data only. No prior study focused specifically on high-risk procedures.

Our data on intensive interventions is more nuanced than that for other outcomes and demonstrates the complexity of decision-making and treatment for patients living with ADRD. Although patients with ADRD have higher burdens of comorbid conditions as shown by higher Elixhauser scores and more frailty, they are less likely to have interventions overall during the index admission and more likely to have them within 90 days when considering death as a competing risk. At both time points, patients with ADRD are more likely to have feeding tube placement. Prolonged intubation is more likely at 90 days as well. It is likely that code status and other discussions regarding end-of-life care impact the use of intensive interventions, but the causes underlying the discrepancy during the index admission and at 90 days are unclear.

The increased likelihood of feeding tube placement in patients with ADRD is particularly noteworthy. Feeding tube placement in patients with advanced dementia has not been found to confer improved survival, quality of life, nutritional status, or caregiver outcomes [33, 34]. To the contrary, feeding tube placement in such cases is associated with a variety of adverse outcomes, such as higher rates of restraint use and healthcare utilization, and is contrary to the position of the American Geriatrics Society [35]. Nevertheless, we cannot make any conclusions about feeding tube appropriateness for several reasons: (1) our analysis does not take into account dementia severity, (2) literature on feeding tubes in generic patients with ADRD cannot be extrapolated directly to their use in surgical patients, and (3) our analysis cannot determine the duration of use.

Importantly, although we did not explicitly measure dementia severity, the cFI index used to measure frailty may also be used to determine moderate-to-severe ADRD using a cutoff of 0.280 or higher [36]. We used the same cFI instrument with a cutoff of 0.250 or higher to dichotomize frailty. Given the similarity of cutoffs, as a first approximation, one could substitute moderate to severe ADRD in place of ADRD with moderate to severe frailty to reframe our results.

Our data and analysis are advantaged by a large sample size, a rich nationwide dataset, inclusion of important covariates such as frailty, and the inclusion of patient-centered outcomes such as time-at-home ratio and intensive interventions. There are, however, several important limitations. First, although we included dual Medicare/Medicaid eligibility as a single measure of socioeconomic status, Medicare data contain little information on social determinants of health, which may be associated with ADRD status and surgical outcomes. Second, we did not have a no-surgery comparison group for a given surgical indication. As a result, our data is unable to answer whether no surgery is better than surgery for ADRD patients in certain situations. Third, we could not analyze patient-reported outcomes, such as physical function and quality of life, which are important in decision-making. Our analysis includes multiple comparisons, which raises the possibility of false positive findings. We have not performed any adjustment for multiple comparisons because following strict cut-offs on significance can cloud some of the nuances of the analysis, especially in exploratory analyses such as this one. This decision is in line with expert guidance, and we acknowledge the need for additional study with prespecified hypotheses for confirmation of our findings [37]. Last, our study is based on Medicare FFS claims data, and extrapolation to other types of patients (e.g., those with Medicare Advantage or without medical insurance) should be done with caution.

## 5 | Conclusions

Patients living with ADRD and undergoing high-risk inpatient surgery are more likely to have inpatient complications, 30- and 90-day mortality, discharge to a higher level of care, prolonged SNF stay, and lower likelihood of discharge home. Intensive interventions during the index admission are less likely except for feeding tube placement, which is more likely. Our findings should be used to inform decision-making for patients living with ADRD.

## Supplementary Material

Supplementary Material **Data S1:** Supporting Information.

Additional supporting information can be found online in the Supporting Information section.

## Figures and Tables

**TABLE 1 |:** Characteristics of patients undergoing high-risk surgery.

	Total population		Without dementia		With dementia		*p*
	*N*	%	*N*	%	*N*	%	
Age, mean (median)	78.0 (76.9)	—	77.0 (75.7)	—	84.0 (84.6)	—	<0.0001
Sex							<0.0001
Male	392,940	45.7	349,370	47.5	43,570	34.9	
Female	466,630	54.3	385,378	52.5	81,252	65.1	
Race							<0.0001
Non-Hispanic White	738,145	85.9	633,663	86.2	104,482	83.7	
Non-Hispanic Black	52,803	6.1	42,666	5.8	10,137	8.1	
Hispanic	36,222	4.2	29,822	4.1	6400	5.1	
Asian/Pacific Islander	13,971	1.6	11,851	1.6	2120	1.7	
Other	5288	0.6	4645	0.6	643	0.5	
Native American	4699	0.6	4017	0.6	682	0.6	
Unknown	8442	1.0	8084	1.1	358	0.3	
Comorbidities							
Diabetes, uncomplicated	264,527	30.8	225,697	30.7	38,830	31.1	0.0057
Hypertension, uncomplicated	693,805	80.7	590,181	80.3	103,624	83.0	<0.0001
Renal failure	244,277	28.4	200,661	27.3	43,616	34.9	<0.0001
COPD	279,061	32.5	238,790	32.5	40,271	32.3	0.0985
Congestive heart failure	273,287	31.8	228,268	31.1	45,019	36.1	<0.0001
Elixhauser index score, mean (median)	13.6 (12.0)	—	13.2 (12.0)		15.8 (15.0)		<0.0001
High risk surgery count on cohort entry date							<0.0001
1	654,017	76.1	542,494	73.8	111,523	89.4	
> 1	205,553	23.9	192,254	26.2	13,299	10.7	
High risk surgery admitting source							< 0.0001
Home	761,901	88.6	659,556	89.8	102,345	82.0	
Facility	24,205	2.8	12,915	1.8	11,290	9.0	
Other Hospital	71,558	8.3	60,694	8.3	10,864	8.7	
Hospice	55	0.0	38	0.0	17	0.0	
Unknown	1851	0.2	1545	0.2	306	0.2	
Dual eligibility							< 0.0001
Yes	142,947	16.6	101,043	13.8	41,904	33.6	
No	716,623	83.4	633,705	86.2	82,918	66.4	
Frail							< 0.0001
Yes	191,255	22.3	137,203	18.7	54,052	43.3	
No	668,315	77.7	597,545	81.3	70,770	56.7	

**TABLE 2 |:** Procedure characteristics and discharge destination

	Total population		Without dementia		With dementia		*p*
Procedure risk, mean (median)^a^	4.3 (2.4)		4.4 (2.4)		3.6 (1.9)		< 0.0001
Admission urgency							< 0.0001
Urgent/Emergent	400,140	46.6	309,412	42.1	90,728	72.7	
Elective	459,430	53.4	425,336	57.9	34,094	27.3	
High Risk surgery discharge status							< 0.01
Home	416,454	48.4	397,560	54.1	18,894	15.1	
Facility	398,543	46.4	303,076	41.2	95,467	76.5	
Hospice	12,597	1.5	7741	1.1	4856	3.9	
Other	1094	0.1	915	0.1	179	0.1	
Expired	30,882	3.6	25,456	3.5	5426	4.3	

a See Section 2.1 for details; defined as the crude inpatient mortality of the procedure in a population of Medicare beneficiaries.

**TABLE 3 |:** Most common high-risk surgeries among patients with and without ADRD.

Non-ADRD ICD-10	NON-ADRD	Count (%)	ADRD ICD-10	Procedure	Count (%)
02RF38Z	Replacement of aortic valve with zooplastic, PERC approach	44,496 (6.1)	0QS706Z	Reposition left upper femur with intramed fix, open approach	8598 (6.9)
021109W	Bypass 2 COR art from aorta with autol vein, open approach	28,375 (3.9)	0QS606Z	Reposition right upper femur with intramed fix, open approach	8326 (6.7)
02RF08Z	Replacement of aortic valve with zooplastic, open approach	20,075 (2.7)	0QS704Z	Reposition left upper femur with int. fix, open approach	6909 (5.5)
0DTF0ZZ	Resection of right large intestine, open approach	18,816 (2.6)	0QS604Z	Reposition right upper femur with int. fix, open approach	6842 (5.5)
021009W	Bypass 1 COR art from aorta with autol vein, open approach	18,469 (2.5)	02RF38Z	Replacement of aortic valve with zooplastic, PERC approach	4220 (3.4)

**TABLE 4 |:** Adjusted and unadjusted outcomes after high-risk surgery in patients with and without ADRD.

	Unadjusted			Adjusted* (ORs or mean difference, 95% CI)		
	Without dementia	With dementia	*p*	Without dementia	With dementia	*p*
	*N* (%)	*N* (%)				
30-day mortality	41,908 (5.7)	15,676 (12.6)	< 0.0001	REF	1.58 (1.54, 1.62)	< 0.0001
90-day mortality	68,550 (9.3)	28,424 (22.8)	< 0.0001	REF	1.82 (1.78, 1.85)	< 0.0001
Inpatient complications	283,053 (38.5)	64,364 (51.6)	< 0.0001	REF	1.19 (1.17, 1.20)	< 0.0001
Prolonged SNF stay^a^ (*n* = 269,458)	2740 (1.4)	2793 (3.7)	< 0.0001	REF	1.80 (1.69, 1.91)	< 0.0001
Hospital LOS during high risk surgery encounter in days, mean (SD)	6.1 (6.3)	5.9 (6.8)	< 0.0001	REF	−0.22 (−0.27, −0.17)	< 0.001
Discharge to home after high risk surgery^b^ (*n* = 761,901)	370,736 (56.2)	17,191 (16.8)	< 0.0001	REF	0.44 (0.43, 0.50)	< 0.0001
Discharge to higher level of care^c^ (*n* = 759,753)	268,738 (41.3)	81,823 (75.1)	< 0.0001	REF	1.49 (1.44, 1.53)	< 0.0001
Home-time ratio, mean (SD)^d^ (*n* = 761,891)	73.0 (31.3)	46.6 (34.5)	< 0.0001	REF	−9.98 (−10.25, −9.71)	< 0.0001
Any intensive intervention during index encounter	34,499 (4.7)	48,409 (3.9)	< 0.001	REF	0.85 (0.82, 0.88)	< 0.001
Prolonged intubation during index encounter	11,731 (1.6)	1498 (1.2)	< 0.001	REF	0.70 (0.66, 0.74)	< 0.001
Prolonged renal replacement therapy during index encounter^e^ (*n* = 838,182)	1508 (0.2)	100 (0.1)	< 0.001	REF	0.41 (0.33, 0.51)	< 0.001
ECMO during index encounter	437 (0.1)	12 (0.0)	< 0.001	REF	0.33 (0.19, 0.60)	< 0.001
Tracheostomy during index encounter^f^ (*N* = 857,621)	6739 (0.9)	682 (0.6)	< 0.001	REF	0.63 (0.58, 0.69)	< 0.001
Cardiopulmonary resuscitation during index encounter	14,186 (1.9)	1280 (1.0)	< 0.001	REF	0.64 (0.60, 0.69)	< 0.001
Feeding tube during index encounter^g^ (*n* = 853,217)	11,971 (1.6)	2575 (2.1)	< 0.001	REF	1.22 (1.17, 1.28)	< 0.001
90-day any intensive intervention^h^ (*n* = 784,528)	43,434 (6.4)	6684 (6.7)	< 0.001	REF	0.98 (0.95, 1.01)	0.15
90-day prolonged intubation^h^ (*n* = 771,556)	14,162 (2.1)	2041 (2.1)	0.80	REF	0.83 (0.79, 0.88)	< 0.001
90-day prolonged renal replacement therapy^i^ (*n* = 748,621)	2239 (0.3)	169 (0.2)	< 0.001	REF	0.46 (0.39, 0.55)	< 0.001
90-day ECMO^h^ (*n* = 762,988)	485 (0.1)	13 (0.0)	< 0.001	REF	0.35 (0.20, 0.61)	< 0.001
90-day tracheostomy^j^ (*n* = 764,619)	7461 (1.1)	805 (0.8)	< 0.001	REF	0.71 (0.65, 0.77)	< 0.001
90-day cardiopulmonary resuscitation^h^ (*n* = 770,932)	17,933 (2.7)	1748 (1.8)	< 0.001	REF	0.71 (0.68, 0.76)	< 0.001
90-day feeding tube^k^ (*n* = 764,788)	15,571 (2.3)	3652 (3.8)	< 0.001	REF	1.41 (1.35, 1.47)	< 0.001

a Skilled nursing facility (SNF) admission over or equal to 100 days. Analysis restricted to patients who had an SNF stay within 3 days after discharge from index encounter and were discharged before September 20, 2018, and with 4 months of continuous Medicare FFS enrollment after discharge (or continuous enrollment till death) to ensure everyone has at least 103 follow-up days.

b Analysis restricted to patients admitted from home.

c Patients who were admitted from the highest level of care (hospital level) were removed from the analysis.

d Analysis restricted to patients admitted from home and home time-ratio between 0 and 1.

e Patients with any ICD-10-Dx code Z99.2 during the index admission before the date of surgery or within 1 year before the index admission were removed from the analysis.

f Patients with any ICD-10-Dx code Z93.0 or Z43.0 during the index admission before the date of surgery or within 1 year before the index admission were removed from the analysis.

g Patients with any ICD-10-Dx code Z93.1 or Z43.1 during the index admission before the date of surgery or within 1 year before the index admission were removed from the analysis.

h Patients who died within 90 days of the index procedure and without the event of interest were removed from the analysis.

i Patients who died within 90 days of the index procedure and without the event of interest were removed from the analysis. Patients with any ICD-10-Dx code Z99.2 during the index admission before the date of surgery or within 1 year before the index admission were removed from the analysis.

j Patients who died within 90 days of the index procedure and without the event of interest were removed from the analysis. Patients with any ICD-10-Dx code Z93.0 or Z43.0 during the index admission before the date of surgery or within 1 year before the index admission were removed from the analysis.

k Patients who died within 90 days of the index procedure and without the event of interest were removed from the analysis. Patients with any ICD-10-Dx code Z93.1 or Z43.1 during the index admission before the date of surgery or within 1 year before the index admission were removed from the analysis.

## Summary

- Key points
  - High-risk surgeries, especially orthopedic and cardiac procedures, are common among older patients with dementia.
  - Mortality, postoperative complications, discharge to home and higher levels of care, time-at-home ratio, and risk of prolonged skilled nursing facility are poor among patients with ADRD undergoing high-risk surgery and worse than among those without ADRD.
- Why does this paper matter?
  - Patients with ADRD undergoing high-risk surgery experience a spectrum of poor outcomes—these data should be incorporated into surgical decision-making to help provide goal-concordant care.
