# Supplementary material for: Patients With Dementia Undergoing High-Risk Inpatient Surgery Have Poor Outcomes
Source: J Am Geriatr Soc. Author manuscript; Available in PMC 2026 Mar 6. (PMC12965178; doi:10.1111/jgs.70091)
Supplement: Supplementary Material — Data S1: Supporting Information. [file NIHMS2144815-supplement-Supplementary_Material.pdf]

Supplemental Figure 1

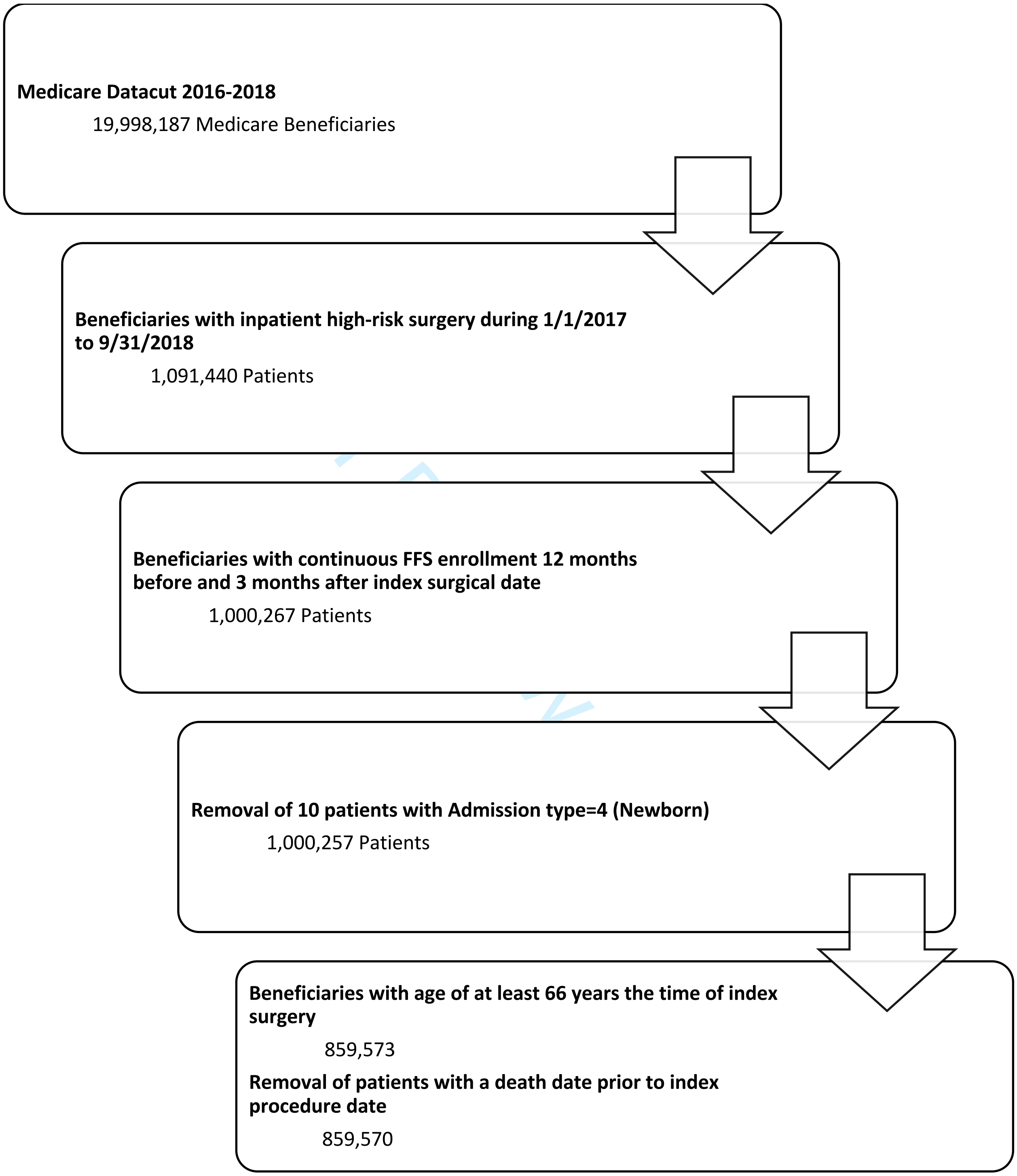

Supplementary Table 1. High-risk procedures classified by dementia status

ICD-10 code	ICD-10 Description	All N	All %	ADRD No N	ADRD No %	ADRD Yes N	ADRD Yes %
02RF38Z	Replacement of aortic valve with zooplastic, perc approach	48,716	5.7	44,496	6.1	4,220	3.4
021109W	Bypass 2 cor art from aorta with autol vn, open approach	29,280	3.4	28,375	3.9	905	0.7
02RF08Z	Replacement of aortic valve with zooplastic, open approach	20,610	2.4	20,075	2.7	535	0.4
0DTF0ZZ	Resection of right large intestine, open approach	20,831	2.4	18,816	2.6	2,015	1.6
021009W	Bypass 1 cor art from aorta with autol vn, open approach	19,068	2.2	18,469	2.5	599	0.5
021209W	Bypass 3 cor art from aorta with autol vn, open approach	17,971	2.1	17,411	2.4	560	0.4
0DB80ZZ	Excision of small intestine, open approach	18,102	2.1	16,203	2.2	1,899	1.5
0QS706Z	Reposition left upper femur with intramed fix, open approach	24,689	2.9	16,091	2.2	8,598	6.9
0QS606Z	Reposition r up femur with intramed fix, open approach	23,668	2.8	15,342	2.1	8,326	6.7
0QS704Z	Reposition left upper femur with int fix, open approach	21,420	2.5	14,511	2.0	6,909	5.5
0QS604Z	Reposition right upper femur with int fix, open approach	21,235	2.5	14,393	2.0	6,842	5.5
0DTF4ZZ	Resection of right large intestine, perc endo approach	14,053	1.6	13,151	1.8	902	0.7
0RG20A0	Fusion 2-6 c jt w intbd fus dev, ant appr a col, open	13,553	1.6	13,132	1.8	421	0.3
0DN80ZZ	Release small intestine, open approach	13,944	1.6	12,456	1.7	1,488	1.2
0WUF0JZ	Supplement abdominal wall with synth sub, open approach	12,308	1.4	11,654	1.6	654	0.5
0DTN0ZZ	Resection of sigmoid colon, open approach	12,647	1.5	11,548	1.6	1,099	0.9
0WQF0ZZ	Repair abdominal wall, open approach	11,794	1.4	10,821	1.5	973	0.8
0SPC0JZ	Removal of synth sub from r knee jt, open approach	10,630	1.2	10,067	1.4	563	0.5
0FT40ZZ	Resection of gallbladder, open approach	10,581	1.2	9,449	1.3	1,132	0.9
0SPD0JZ	Removal of synth sub from l knee jt, open approach	9,882	1.1	9,350	1.3	532	0.4
04CL0ZZ	Extirpation of matter from l fem art, open approach	10,206	1.2	9,299	1.3	907	0.7
0DBN0ZZ	Excision of sigmoid colon, open approach	9,061	1.1	8,319	1.1	742	0.6
00NW0ZZ	Release cervical spinal cord, open approach	8,699	1.0	8,221	1.1	478	0.4
02RG08Z	Replacement of mitral valve with zooplastic, open approach	8,481	1.0	8,166	1.1	315	0.3
04CK0ZZ	Extirpation of matter from r fem art, open approach	8,909	1.0	8,133	1.1	776	0.6
0DTN4ZZ	Resection of sigmoid colon, percutaneous endoscopic approach	8,204	1.0	7,841	1.1	363	0.3
0D1N0Z4	Bypass sigmoid colon to cutaneous, open approach	8,609	1.0	7,422	1.0	1,187	1.0
0SRS0J9	Replace l hip jt, femoral w synth sub, cement, open	11,260	1.3	7,043	1.0	4,217	3.4
0SRS0JZ	Replace of l hip jt, femoral with synth sub, open approach	11,044	1.3	6,995	1.0	4,049	3.2
02100Z9	Bypass 1 cor art from l int mammary, open approach	7,103	0.8	6,879	0.9	224	0.2
00C40ZZ	Extirpation of matter from cran subdur spc, open approach	8,530	1.0	6,822	0.9	1,708	1.4
0SRR0J9	Replace r hip jt, femoral w synth sub, cement, open	10,656	1.2	6,676	0.9	3,980	3.2
0SRR0JZ	Replace of r hip jt, femoral with synth sub, open approach	10,391	1.2	6,673	0.9	3,718	3.0
0SRS0JA	Replace l hip jt, femoral w synth sub, uncement, open	10,273	1.2	6,494	0.9	3,779	3.0
0RG2071	Fusion 2-6 c jt w autol sub, post appr p col, open	6,746	0.8	6,319	0.9	427	0.3
0SRR0JA	Replace r hip jt, femoral w synth sub, uncement, open	9,777	1.1	6,293	0.9	3,484	2.8
0BQT4ZZ	Repair diaphragm, percutaneous endoscopic approach	5,623	0.7	5,416	0.7	207	0.2
02RX0JZ	Replacement of thor aorta asc with synth sub, open approach	5,434	0.6	5,290	0.7	144	0.1
0QSB04Z	Reposition right lower femur with int fix, open approach	6,834	0.8	5,155	0.7	1,679	1.3

0DB60ZZ	Excision of stomach, open approach	5,498	0.6	5,124	0.7	374	0.3
0SP90JZ	Removal of synthetic substitute from r hip jt, open approach	5,505	0.6	5,078	0.7	427	0.3
021309W	Bypass 4+ cor art from aorta with autol vn, open approach	5,123	0.6	5,007	0.7	116	0.1
0DBB0ZZ	Excision of ileum, open approach	5,120	0.6	4,773	0.6	347	0.3
0RG7071	Fusion 2-7 t jt w autol sub, post appr p col, open	4,900	0.6	4,640	0.6	260	0.2
0QSC04Z	Reposition left lower femur with int fix, open approach	5,995	0.7	4,574	0.6	1,421	1.1
0SPB0JZ	Removal of synthetic substitute from l hip jt, open approach	4,912	0.6	4,534	0.6	378	0.3
00B70ZZ	Excision of cerebral hemisphere, open approach	4,717	0.5	4,476	0.6	241	0.2
0DTJ0ZZ	Resection of appendix, open approach	4,364	0.5	4,095	0.6	269	0.2
07TP0ZZ	Resection of spleen, open approach	4,285	0.5	4,074	0.6	211	0.2
0DBA0ZZ	Excision of jejunum, open approach	4,419	0.5	4,012	0.5	407	0.3
0SRS01A	Replace l hip jt, femoral w metal, uncement, open	6,121	0.7	3,944	0.5	2,177	1.7
0SRR01A	Replace r hip jt, femoral w metal, uncement, open	5,810	0.7	3,839	0.5	1,971	1.6
00B00ZZ	Excision of brain, open approach	3,953	0.5	3,722	0.5	231	0.2
0DTG0ZZ	Resection of left large intestine, open approach	3,967	0.5	3,586	0.5	381	0.3
00NX0ZZ	Release thoracic spinal cord, open approach	3,653	0.4	3,427	0.5	226	0.2
0TT00ZZ	Resection of right kidney, open approach	3,546	0.4	3,371	0.5	175	0.1
02UG0JZ	Supplement mitral valve with synth sub, open approach	3,286	0.4	3,227	0.4	59	0.0
0TT10ZZ	Resection of left kidney, open approach	3,363	0.4	3,207	0.4	156	0.1
0211093	Bypass 2 cor art from cor art with autol vn, open approach	3,234	0.4	3,134	0.4	100	0.1
0SRS019	Replace l hip jt, femoral w metal, cement, open	4,852	0.6	3,087	0.4	1,765	1.4
0D1L0Z4	Bypass transverse colon to cutaneous, open approach	3,534	0.4	3,067	0.4	467	0.4
0TTB0ZZ	Resection of bladder, open approach	3,164	0.4	3,059	0.4	105	0.1
0W9D00Z	Drainage of pericardial cavity with drain dev, open approach	3,314	0.4	3,030	0.4	284	0.2
0SRR019	Replace r hip jt, femoral w metal, cement, open	4,678	0.5	3,017	0.4	1,661	1.3
0BUT4JZ	Supplement diaphragm with synth sub, perc endo approach	2,877	0.3	2,749	0.4	128	0.1
0DT80ZZ	Resection of small intestine, open approach	3,088	0.4	2,727	0.4	361	0.3
0SR90J9	Replace of r hip jt with synth sub, cement, open approach	3,014	0.4	2,708	0.4	306	0.2
0QH706Z	Insertion of intramed fix into l up femur, open approach	3,874	0.5	2,613	0.4	1,261	1.0
0W9B40Z	Drainage of l pleural cav with drain dev, perc endo approach	2,797	0.3	2,587	0.4	210	0.2
0DB90ZZ	Excision of duodenum, open approach	2,662	0.3	2,572	0.3	90	0.1
0DNE0ZZ	Release large intestine, open approach	2,758	0.3	2,540	0.3	218	0.2
0DQ80ZZ	Repair small intestine, open approach	2,753	0.3	2,499	0.3	254	0.2
0Y6H0Z1	Detachment at right lower leg, high, open approach	3,072	0.4	2,435	0.3	637	0.5
0QH606Z	Insertion of intramed fix into r up femur, open approach	3,671	0.4	2,418	0.3	1,253	1.0
0DU907Z	Supplement duodenum with autol sub, open approach	2,855	0.3	2,413	0.3	442	0.4
0Y6J0Z1	Detachment at left lower leg, high, open approach	2,926	0.3	2,366	0.3	560	0.4
02580ZZ	Destruction of conduction mechanism, open approach	2,343	0.3	2,299	0.3	44	0.0
0SRB0J9	Replace of l hip jt with synth sub, cement, open approach	2,574	0.3	2,298	0.3	276	0.2
02VW3DZ	Restrict of thor aorta desc with intralum dev, perc approach	2,460	0.3	2,289	0.3	171	0.1
0QS804Z	Reposition right femoral shaft with int fix, open approach	3,115	0.4	2,277	0.3	838	0.7
0BTF4ZZ	Resection of right lower lung lobe, perc endo approach	2,328	0.3	2,263	0.3	65	0.1
0BTC0ZZ	Resection of right upper lung lobe, open approach	2,304	0.3	2,246	0.3	58	0.0
00B10ZZ	Excision of cerebral meninges, open approach	2,336	0.3	2,164	0.3	172	0.1

0DTE0ZZ	Resection of large intestine, open approach	2,473	0.3	2,149	0.3	324	0.3
02100A9	Bypass 1 cor art from l int mammary w autol art, open	2,193	0.3	2,111	0.3	82	0.1
0SRR01Z	Replacement of r hip jt, femoral with metal, open approach	3,092	0.4	2,075	0.3	1,017	0.8
0DBL0ZZ	Excision of transverse colon, open approach	2,226	0.3	2,040	0.3	186	0.1
02RF0JZ	Replacement of aortic valve with synth sub, open approach	2,080	0.2	2,026	0.3	54	0.0
0QS904Z	Reposition left femoral shaft with int fix, open approach	2,794	0.3	1,996	0.3	798	0.6
04QK0ZZ	Repair right femoral artery, open approach	2,149	0.2	1,980	0.3	169	0.1
0SRS01Z	Replacement of l hip jt, femoral with metal, open approach	3,101	0.4	1,965	0.3	1,136	0.9
0QB10ZZ	Excision of sacrum, open approach	3,658	0.4	1,952	0.3	1,706	1.4
009400Z	Drainage of cran subdur spc with drain dev, open approach	2,523	0.3	1,948	0.3	575	0.5
0D160ZA	Bypass stomach to jejunum, open approach	2,099	0.2	1,936	0.3	163	0.1
0FB00ZX	Excision of liver, open approach, diagnostic	2,042	0.2	1,934	0.3	108	0.1
0Y6C0Z3	Detachment at right upper leg, low, open approach	2,981	0.3	1,834	0.2	1,147	0.9
0210099	Bypass 1 cor art from l int mammary w autol vn, open	1,869	0.2	1,809	0.2	60	0.0
0BTG0ZZ	Resection of left upper lung lobe, open approach	1,845	0.2	1,801	0.2	44	0.0
0DTL0ZZ	Resection of transverse colon, open approach	1,984	0.2	1,785	0.2	199	0.2
0DBM0ZZ	Excision of descending colon, open approach	1,947	0.2	1,764	0.2	183	0.1
0W3Q7ZZ	Control bleeding in respiratory tract, via opening	2,051	0.2	1,753	0.2	298	0.2
0TQB0ZZ	Repair bladder, open approach	1,909	0.2	1,737	0.2	172	0.1
0Y6D0Z3	Detachment at left upper leg, low, open approach	2,808	0.3	1,735	0.2	1,073	0.9
04CM0ZZ	Extirpation of matter from r popl art, open approach	1,956	0.2	1,720	0.2	236	0.2
0RG6071	Fusion thor jt w autol sub, post appr p col, open	1,806	0.2	1,702	0.2	104	0.1
04CN0ZZ	Extirpation of matter from l popl art, open approach	1,964	0.2	1,695	0.2	269	0.2
0DNB0ZZ	Release ileum, open approach	1,862	0.2	1,661	0.2	201	0.2
0WPF0JZ	Removal of synthetic substitute from abd wall, open approach	1,766	0.2	1,660	0.2	106	0.1
00B00ZX	Excision of brain, open approach, diagnostic	1,817	0.2	1,652	0.2	165	0.1
0BTJ4ZZ	Resection of left lower lung lobe, perc endo approach	1,642	0.2	1,601	0.2	41	0.0
009600Z	Drainage of cerebral ventricle with drain dev, open approach	1,768	0.2	1,560	0.2	208	0.2
0DTH0ZZ	Resection of cecum, open approach	1,711	0.2	1,555	0.2	156	0.1
00B70ZX	Excision of cerebral hemisphere, open approach, diagnostic	1,655	0.2	1,522	0.2	133	0.1
0W9D0ZZ	Drainage of pericardial cavity, open approach	1,652	0.2	1,514	0.2	138	0.1
0DB84ZZ	Excision of small intestine, perc endo approach	1,531	0.2	1,426	0.2	105	0.1
041L0JL	Bypass l fem art to poplit art with synth sub, open approach	1,534	0.2	1,410	0.2	124	0.1
041K0JL	Bypass r fem art to poplit art with synth sub, open approach	1,531	0.2	1,408	0.2	123	0.1
0QS806Z	Reposition r femur shaft with intramed fix, open approach	1,835	0.2	1,387	0.2	448	0.4
0FBG4ZZ	Excision of pancreas, percutaneous endoscopic approach	1,435	0.2	1,384	0.2	51	0.0
0DQ60ZZ	Repair stomach, open approach	1,667	0.2	1,352	0.2	315	0.3
0DQ90ZZ	Repair duodenum, open approach	1,570	0.2	1,346	0.2	224	0.2
0BTF0ZZ	Resection of right lower lung lobe, open approach	1,395	0.2	1,344	0.2	51	0.0
02C00ZZ	Extirpation of matter from 1 cor art, open approach	1,390	0.2	1,331	0.2	59	0.0
0QS906Z	Reposition l femur shaft with intramed fix, open approach	1,687	0.2	1,292	0.2	395	0.3
0RG1071	Fusion cerv jt w autol sub, post appr p col, open	1,387	0.2	1,263	0.2	124	0.1
02RG0JZ	Replacement of mitral valve with synth sub, open approach	1,283	0.1	1,248	0.2	35	0.0
0DBF0ZZ	Excision of right large intestine, open approach	1,374	0.2	1,246	0.2	128	0.1

0DQ64ZZ	Repair stomach, percutaneous endoscopic approach	1,343	0.2	1,208	0.2	135	0.1
00N00ZZ	Release brain, open approach	1,299	0.2	1,189	0.2	110	0.1
0DBK0ZZ	Excision of ascending colon, open approach	1,294	0.2	1,179	0.2	115	0.1
X2RF032	Replace aort valve w zoopl rapid depl, open, new tech 2	1,202	0.1	1,172	0.2	30	0.0
0DBH0ZZ	Excision of cecum, open approach	1,300	0.2	1,170	0.2	130	0.1
0Y6H0Z2	Detachment at right lower leg, mid, open approach	1,430	0.2	1,151	0.2	279	0.2
0DTG4ZZ	Resection of left large intestine, perc endo approach	1,213	0.1	1,150	0.2	63	0.1
0RG70K1	Fusion 2-7 t jt w nonaut sub, post appr p col, open	1,226	0.1	1,136	0.2	90	0.1
00940ZZ	Drainage of intracranial subdural space, open approach	1,396	0.2	1,085	0.1	311	0.2
0BTJ0ZZ	Resection of left lower lung lobe, open approach	1,126	0.1	1,083	0.1	43	0.0
0Y6J0Z2	Detachment at left lower leg, mid, open approach	1,324	0.2	1,067	0.1	257	0.2
0BNK4ZZ	Release right lung, percutaneous endoscopic approach	1,167	0.1	1,061	0.1	106	0.1
0PS304Z	Reposition cervical vertebra with int fix, open approach	1,247	0.1	1,051	0.1	196	0.2
04QL0ZZ	Repair left femoral artery, open approach	1,123	0.1	1,047	0.1	76	0.1
0DTK0ZZ	Resection of ascending colon, open approach	1,138	0.1	1,036	0.1	102	0.1
041K09L	Bypass r fem art to poplit art with autol vn, open approach	1,093	0.1	1,020	0.1	73	0.1
0BBC0ZZ	Excision of right upper lung lobe, open approach	1,047	0.1	1,009	0.1	38	0.0
00C70ZZ	Extirpation of matter from cereb hemis, open approach	1,145	0.1	998	0.1	147	0.1
0FB10ZZ	Excision of right lobe liver, open approach	1,019	0.1	996	0.1	23	0.0
00BC0ZZ	Excision of cerebellum, open approach	1,023	0.1	992	0.1	31	0.0
0Y6H0Z3	Detachment at right lower leg, low, open approach	1,203	0.1	981	0.1	222	0.2
041L09L	Bypass l fem art to poplit art with autol vn, open approach	1,037	0.1	975	0.1	62	0.0
0FBG0ZZ	Excision of pancreas, open approach	1,000	0.1	973	0.1	27	0.0
0Y6J0Z3	Detachment at left lower leg, low, open approach	1,182	0.1	941	0.1	241	0.2
03C80ZZ	Extirpation of matter from l brach art, open approach	1,173	0.1	938	0.1	235	0.2
0DBE0ZZ	Excision of large intestine, open approach	1,054	0.1	929	0.1	125	0.1
02RF0KZ	Replacement of aortic valve with nonaut sub, open approach	951	0.1	919	0.1	32	0.0
0QSB06Z	Reposition r low femur with intramed fix, open approach	1,303	0.2	915	0.1	388	0.3
0BQT0ZZ	Repair diaphragm, open approach	963	0.1	894	0.1	69	0.1
0DNA0ZZ	Release jejunum, open approach	1,013	0.1	877	0.1	136	0.1
0DTB0ZZ	Resection of ileum, open approach	945	0.1	858	0.1	87	0.1
0DTP0ZZ	Resection of rectum, open approach	972	0.1	853	0.1	119	0.1
0QSC06Z	Reposition left lower femur with intramed fix, open approach	1,149	0.1	842	0.1	307	0.2
0BBG0ZZ	Excision of left upper lung lobe, open approach	840	0.1	816	0.1	24	0.0
0DBP0ZZ	Excision of rectum, open approach	897	0.1	798	0.1	99	0.1
0DTM0ZZ	Resection of descending colon, open approach	890	0.1	797	0.1	93	0.1
03C70ZZ	Extirpation of matter from r brach art, open approach	885	0.1	711	0.1	174	0.1
0W3P0ZZ	Control bleeding in gastrointestinal tract, open approach	784	0.1	697	0.1	87	0.1
0BBF0ZX	Excision of right lower lung lobe, open approach, diagnostic	712	0.1	687	0.1	25	0.0
0QS504Z	Reposition left acetabulum with int fix, open approach	789	0.1	679	0.1	110	0.1
0BNL4ZZ	Release left lung, percutaneous endoscopic approach	738	0.1	678	0.1	60	0.0
03LG0CZ	Occlusion of intracran art with extralum dev, open approach	687	0.1	665	0.1	22	0.0
0DQ70ZZ	Repair stomach, pylorus, open approach	750	0.1	662	0.1	88	0.1
02VX3DZ	Restrict of thor aorta asc with intralum dev, perc approach	711	0.1	655	0.1	56	0.0

0QS404Z	Reposition right acetabulum with int fix, open approach	737	0.1	638	0.1	99	0.1
0BBF0ZZ	Excision of right lower lung lobe, open approach	658	0.1	633	0.1	25	0.0
0PB00ZZ	Excision of sternum, open approach	674	0.1	629	0.1	45	0.0
00C00ZZ	Extirpation of matter from brain, open approach	711	0.1	627	0.1	84	0.1
0BNL0ZZ	Release left lung, open approach	660	0.1	618	0.1	42	0.0
0DBG0ZZ	Excision of left large intestine, open approach	660	0.1	590	0.1	70	0.1
0BNK0ZZ	Release right lung, open approach	651	0.1	590	0.1	61	0.0
0Y6M0Z0	Detachment at right foot, complete, open approach	688	0.1	578	0.1	110	0.1
02RX08Z	Replacement of thor aorta asc with zooplastic, open approach	569	0.1	*	*	*	*
04U00JZ	Supplement abdominal aorta with synth sub, open approach	573	0.1	540	0.1	33	0.0
0DU947Z	Supplement duodenum with autol sub, perc endo approach	603	0.1	525	0.1	78	0.1
0Y6N0Z0	Detachment at left foot, complete, open approach	652	0.1	523	0.1	129	0.1
0Y6F0ZZ	Detachment at right knee region, open approach	692	0.1	521	0.1	171	0.1
0DU607Z	Supplement stomach with autol sub, open approach	600	0.1	517	0.1	83	0.1
0DB70ZZ	Excision of stomach, pylorus, open approach	553	0.1	515	0.1	38	0.0
0Y6G0ZZ	Detachment at left knee region, open approach	676	0.1	497	0.1	179	0.1
0Y6C0Z2	Detachment at right upper leg, mid, open approach	725	0.1	458	0.1	267	0.2
0BBJ0ZX	Excision of left lower lung lobe, open approach, diagnostic	471	0.1	453	0.1	18	0.0
0DTA0ZZ	Resection of jejunum, open approach	511	0.1	452	0.1	59	0.0
0Y6D0Z2	Detachment at left upper leg, mid, open approach	695	0.1	447	0.1	248	0.2
03VG0CZ	Restrict of intracran art with extralum dev, open approach	440	0.1	416	0.1	24	0.0
0Y6D0Z1	Detachment at left upper leg, high, open approach	687	0.1	412	0.1	275	0.2
02RJ08Z	Replacement of tricusp valve with zooplastic, open approach	429	0.0	412	0.1	17	0.0
0FT10ZZ	Resection of right lobe liver, open approach	408	0.0	*	*	*	*
0DQB0ZZ	Repair ileum, open approach	418	0.0	390	0.1	28	0.0
0Y6C0Z1	Detachment at right upper leg, high, open approach	618	0.1	381	0.1	237	0.2
0DT60ZZ	Resection of stomach, open approach	401	0.0	380	0.1	21	0.0
0QBS0ZZ	Excision of coccyx, open approach	660	0.1	377	0.1	283	0.2
02RW0JZ	Replacement of thor aorta desc with synth sub, open approach	381	0.0	370	0.1	11	0.0
04Q00ZZ	Repair abdominal aorta, open approach	378	0.0	359	0.0	19	0.0
04C50ZZ	Extirpation of matter from sup mesent art, open approach	394	0.0	337	0.0	57	0.0
00CC0ZZ	Extirpation of matter from cerebellum, open approach	302	0.0	277	0.0	25	0.0
02VW3EZ	Restrict thor aorta desc w fenestr dev 1 or 2, perc	282	0.0	264	0.0	18	0.0
00C30ZZ	Extirpation of matter from cran epidur spc, open approach	286	0.0	257	0.0	29	0.0
0BNF0ZZ	Release right lower lung lobe, open approach	262	0.0	247	0.0	15	0.0
00970ZZ	Drainage of cerebral hemisphere, open approach	244	0.0	206	0.0	38	0.0
0DU707Z	Supplement stomach, pylorus with autol sub, open approach	239	0.0	197	0.0	42	0.0
0BNJ0ZZ	Release left lower lung lobe, open approach	208	0.0	195	0.0	13	0.0
0WC10ZZ	Extirpation of matter from cranial cavity, open approach	201	0.0	161	0.0	40	0.0
00NC0ZZ	Release cerebellum, open approach	85	0.0	*	*	*	*
041K0JK	Bypass r fem art to b femor a with synth sub, open approach	32	0.0	*	*	*	*

*Values suppressed in accordance with Centers for Medicare and Medicaid Services policy

Supplementary Table 2. Frequency of major complications by dementia status

		All		ADRD No		ADRD Yes	
		N	%	N	%	N	%
ICD-10 Code	ICD-10 Description						
I2699	Other pulmonary embolism without acute cor pulmonale	12,172	1.9336	10,538	2.0314	1,634	1.4754
L7632	Postproc hematoma of skin, subcu following other procedure	3,640	0.5782	3,218	0.6203	422	0.3810
I97611	Postproc hemor of a circ sys org following cardiac bypass	1,595	0.2534	1,543	0.2974	52	0.0470
I82621	Acute embolism and thrombosis of deep veins of r up extrem	1,559	0.2477	1,327	0.2558	232	0.2095
I97638	Postproc hematoma of a circ sys org fol other circ sys proc	1,531	0.2432	1,398	0.2695	133	0.1201
I97618	Postproc hemor of a circ sys org fol other circ sys proc	1,493	0.2372	1,404	0.2707	89	0.0804
K91840	Postproc hemor of a dgstv sys org fol a dgstv sys procedure	1,254	0.1992	1,143	0.2203	111	0.1002
I82611	Acute embolism and thombos of superfic veins of r up extrem	1,198	0.1903	1,039	0.2003	159	0.1436
I82622	Acute embolism and thrombosis of deep veins of l up extrem	1,176	0.1868	986	0.1901	190	0.1716
I82612	Acute embolism and thombos of superfic veins of l up extrem	1,068	0.1697	939	0.1810	129	0.1165
I82C11	Acute embolism and thrombosis of right internal jugular vein	858	0.1363	799	0.1540	59	0.0533
I97418	Intraop hemor/hemtom of circ sys org comp oth circ sys proc	757	0.1203	716	0.1380	41	0.0370
M96840	Postproc hematoma of a ms structure fol a ms sys procedure	746	0.1185	597	0.1151	149	0.1345
K91870	Postproc hematoma of a dgstv sys org fol a dgstv sys proc	713	0.1133	648	0.1249	65	0.0587
L7622	Postproc hemorrhage of skin, subcu following other procedure	707	0.1123	641	0.1236	66	0.0596
K228	Other specified diseases of esophagus	655	0.1041	560	0.1080	95	0.0858
I97630	Postproc hematoma of a circ sys org following a cardiac cath	653	0.1037	607	0.1170	46	0.0415
K9161	Intraop hemor/hemtom of dgstv sys org comp a dgstv sys proc	416	0.0661	370	0.0713	46	0.0415
I97631	Postproc hematoma of a circ sys org following cardiac bypass	410	0.0651	397	0.0765	13	0.0117
I82A11	Acute embolism and thrombosis of right axillary vein	391	0.0621	340	0.0655	51	0.0461
I82B11	Acute embolism and thrombosis of right subclavian vein	373	0.0593	333	0.0642	40	0.0361
I82B12	Acute embolism and thrombosis of left subclavian vein	356	0.0566	306	0.0590	50	0.0451
M96841	Postproc hematoma of a ms structure fol other procedure	356	0.0566	333	0.0642	23	0.0208
G9761	Postp hematoma of a nervous sys org fol a nervous sys proc	333	0.0529	314	0.0605	19	0.0172
I82C12	Acute embolism and thrombosis of left internal jugular vein	308	0.0489	273	0.0526	35	0.0316
I97620	Postproc hemor of a circ sys org following other procedure	275	0.0437	258	0.0497	17	0.0154
I97411	Intraop hemor/hemtom of a circ sys org comp card bypass	272	0.0432	259	0.0499	13	0.0117
I9742	Intraop hemor/hemtom of a circ sys org comp oth procedure	267	0.0424	252	0.0486	15	0.0135

G9751	Postproc hemor of a nervous sys org fol a nervous sys proc	257	0.0408	230	0.0443	27	0.0244
I82A12	Acute embolism and thrombosis of left axillary vein	247	0.0392	211	0.0407	36	0.0325
I82890	Acute embolism and thrombosis of other specified veins	224	0.0356	208	0.0401	16	0.0144
I82613	Acute emblsm and thombos of superfic veins of up extrem, bi	206	0.0327	184	0.0355	22	0.0199
M96830	Postproc hemor of a ms structure fol a ms sys procedure	196	0.0311	153	0.0295	43	0.0388
I82623	Acute embolism and thombos of deep veins of up extrem, bi	186	0.0295	160	0.0308	26	0.0235
K91871	Postproc hematoma of a dgstv sys org fol other procedure	180	0.0286	164	0.0316	16	0.0144
I97610	Postproc hemor of a circ sys org following a cardiac cath	171	0.0272	159	0.0307	12	0.0108
K91841	Postproc hemor of a dgstv sys org following other procedure	170	0.0270	144	0.0278	26	0.0235
T82818A	Embolism due to vascular prosth dev/grft, initial encounter	169	0.0268	151	0.0291	18	0.0163
M96831	Postproc hemor of a ms structure following other procedure	161	0.0256	149	0.0287	12	0.0108
I97621	Postproc hematoma of a circ sys org fol other procedure	160	0.0254	148	0.0285	12	0.0108
J95831	Postproc hemor of a resp sys org following other procedure	158	0.0251	*	*	*	*
I2609	Other pulmonary embolism with acute cor pulmonale	146	0.0232	128	0.0247	18	0.0163
J95830	Postproc hemor of a resp sys org fol a resp sys procedure	142	0.0226	*	*	*	*
M96810	Intraop hemor/hemtom of a ms structure comp a ms sys proc	133	0.0211	104	0.0200	29	0.0262
I82619	Acute embolism and thrombosis of superfic vn unsp up extrem	132	0.0210	115	0.0222	17	0.0154
I2692	Saddle embolus of pulmonary artery w/o acute cor pulmonale	131	0.0208	107	0.0206	24	0.0217
N99820	Postproc hemor of a gu sys org following a gu sys procedure	127	0.0202	*	*	*	*
N99840	Postproc hematoma of a gu sys org fol a gu sys procedure	91	0.0145	*	*	*	*
I82629	Acute embolism and thrombosis of deep vn unsp up extrem	88	0.0140	72	0.0139	16	0.0144
N9961	Intraop hemor/hemtom of a gu sys org comp a gu sys procedure	88	0.0140	*	*	*	*
G9762	Postproc hematoma of a nervous sys org fol other procedure	75	0.0119	*	*	*	*
I2690	Septic pulmonary embolism without acute cor pulmonale	75	0.0119	*	*	*	*
I82290	Acute embolism and thrombosis of other thoracic veins	75	0.0119	*	*	*	*
I97410	Intraoperative hemor/hemtom of a circ sys org comp card cath	75	0.0119	*	*	*	*
J95861	Postproc hematoma of a resp sys org fol other procedure	72	0.0114	*	*	*	*
D7802	Intraop hemor/hemtom of the spleen comp oth procedure	67	0.0106	*	*	*	*
I82C13	Acute embolism and thrombosis of int jugular vein, bilateral	57	0.0091	*	*	*	*
J95860	Postproc hematoma of a resp sys org fol a resp sys procedure	57	0.0091	*	*	*	*
I82210	Acute embolism and thrombosis of superior vena cava	55	0.0087	*	*	*	*
L7631	Postproc hematoma of skin, subcu fol a dermatologic proc	55	0.0087	*	*	*	*
K9162	Intraop hemor/hemtom of a dgstv sys org comp oth procedure	54	0.0086	*	*	*	*

I82C19	Acute embolism and thrombosis of unsp internal jugular vein	53	0.0084	*	*	*	*
N99841	Postproc hematoma of a gu sys org following other procedure	52	0.0083	*	*	*	*
G9731	Intraop hemor/hemtom of a nervous sys org comp nrv sys proc	51	0.0081	*	*	*	*
J9561	Intraop hemor/hemtom of a resp sys org comp resp sys proc	49	0.0078	*	*	*	*
L7602	Intraop hemor/hemtom of skin, subcu comp oth procedure	48	0.0076	*	*	*	*
J9562	Intraop hemor/hemtom of a resp sys org comp oth procedure	45	0.0071	*	*	*	*
M96811	Intraop hemor/hemtom of a ms structure comp oth procedure	44	0.0070	*	*	*	*
G9752	Postproc hemor of a nervous sys org fol other procedure	41	0.0065	*	*	*	*
I8290	Acute embolism and thrombosis of unspecified vein	34	0.0054	22	0.0042	12	0.0108
D7801	Intraop hemor/hemtom of the spleen comp a proc on the spleen	33	0.0052	*	*	*	*
T82817A	Embolism due to cardiac prosth dev/grft, initial encounter	28	0.0044	*	*	*	*
I803	Phlebitis and thrombophlebitis of lower extremities, unsp	26	0.0041	*	*	*	*
I82A19	Acute embolism and thrombosis of unspecified axillary vein	24	0.0038	*	*	*	*
I82601	Acute embolism and thombos unsp veins of r up extrem	23	0.0037	*	*	*	*
I82602	Acute embolism and thombos unsp veins of l up extrem	23	0.0037	*	*	*	*
D7822	Postproc hemorrhage of the spleen following other procedure	22	0.0035	*	*	*	*
I82B13	Acute embolism and thrombosis of subclavian vein, bilateral	22	0.0035	*	*	*	*
I2602	Saddle embolus of pulmonary artery with acute cor pulmonale	21	0.0033	*	*	*	*
I82B19	Acute embolism and thrombosis of unspecified subclavian vein	20	0.0032	*	*	*	*
L7621	Postproc hemor of skin, subcu fol a dermatologic procedure	20	0.0032	*	*	*	*
N99821	Postproc hemor of a gu sys org following other procedure	17	0.0027	*	*	*	*
I82A13	Acute embolism and thrombosis of axillary vein, bilateral	16	0.0025	*	*	*	*
D7832	Postproc hematoma of the spleen following other procedure	13	0.0021	*	*	*	*
N9962	Intraop hemor/hemtom of a gu sys org comp oth procedure	13	0.0021	*	*	*	*
E89810	Postproc hemor of an endo sys org fol an endo sys procedure	11	0.0017	*	*	*	*
E89820	Postproc hematoma of an endo sys org fol an endo sys proc	11	0.0017	*	*	*	*
L7601	Intraop hemor/hemtom of skin, subcu comp a dermatologic proc	11	0.0017	*	*	*	*
G9732	Intraop hemor/hemtom of a nervous sys org comp oth procedure	*	*	*	*	*	*
I8011	Phlebitis and thrombophlebitis of right femoral vein	*	*	*	*	*	*
D7831	Postprocedural hematoma of the spleen fol proc on spleen	*	*	*	*	*	*
E3602	Intraop hemor/hemtom of an endo sys org comp oth procedure	*	*	*	*	*	*
D7821	Postprocedural hemorrhage of the spleen fol proc on spleen	*	*	*	*	*	*

I82603	Acute embolism and thombos unsp veins of up extrem, bi	*	*	*	*	*	*
E3601	Intraop hemor/hemtom of endo sys org comp an endo sys proc	*	*	*	*	*	*
E89821	Postproc hematoma of an endo sys org fol other procedure	*	*	*	*	*	*
I82609	Acute embolism and thombos unsp vn unsp upper extremity	*	*	*	*	*	*
E89811	Postproc hemor of an endo sys org following other procedure	*	*	*	*	*	*
I2601	Septic pulmonary embolism with acute cor pulmonale	*	*	*	*	*	*
I80299	Phlebitis and thombophlb of deep vessels of unsp low extrm	*	*	*	*	*	*
T800XXA	Air embolism fol infusion, tranfs and theraputc inject, init	*	*	*	*	*	*
I8010	Phlebitis and thrombophlebitis of unspecified femoral vein	*	*	*	*	*	*
I80201	Phlbts and thombophlb of unsp deep vessels of r low extrem	*	*	*	*	*	*
I80291	Phlebitis and thombophlb of deep vessels of r low extrem	*	*	*	*	*	*
H9541	Postprocedural hemorrhage of ear/mastd fol proc on ear/mastd	*	*	*	*	*	*
I8012	Phlebitis and thrombophlebitis of left femoral vein	*	*	*	*	*	*
I80203	Phlbts and thombophlb of unsp deep vessels of low extrm, bi	*	*	*	*	*	*
I80209	Phlbts and thombophlb of unsp deep vessels of unsp low extrm	*	*	*	*	*	*
I80211	Phlebitis and thrombophlebitis of right iliac vein	*	*	*	*	*	*
I80221	Phlebitis and thrombophlebitis of right popliteal vein	*	*	*	*	*	*
I80231	Phlebitis and thrombophlebitis of right tibial vein	*	*	*	*	*	*
H59321	Postproc hemor of right eye and adnexa fol other procedure	*	*	*	*	*	*
H59341	Postproc hematoma of right eye and adnexa fol other proc	*	*	*	*	*	*
H59343	Postproc hematoma of eye and adnexa fol other procedure, bi	*	*	*	*	*	*
I8013	Phlebitis and thrombophlebitis of femoral vein, bilateral	*	*	*	*	*	*
I80202	Phlbts and thombophlb of unsp deep vessels of l low extrem	*	*	*	*	*	*
I80222	Phlebitis and thrombophlebitis of left popliteal vein	*	*	*	*	*	*
I80229	Phlebitis and thrombophlebitis of unspecified popliteal vein	*	*	*	*	*	*
I80232	Phlebitis and thrombophlebitis of left tibial vein	*	*	*	*	*	*
I80239	Phlebitis and thrombophlebitis of unspecified tibial vein	*	*	*	*	*	*
I80292	Phlebitis and thombophlb of deep vessels of l low extrem	*	*	*	*	*	*
I80293	Phlebitis and thombophlb of deep vessels of low extrm, bi	*	*	*	*	*	*
I214	Non-st elevation (nstemi) myocardial infarction	12,492	1.9844	10,463	2.0170	2,029	1.8321
I469	Cardiac arrest, cause unspecified	7,038	1.1180	6,038	1.1640	1,000	0.9030
T8111XA	Postprocedural cardiogenic shock, initial encounter	3,340	0.5306	3,204	0.6176	136	0.1228
I21A1	Myocardial infarction type 2	3,217	0.5110	2,594	0.5000	623	0.5625
I462	Cardiac arrest due to underlying cardiac condition	1,033	0.1641	968	0.1866	65	0.0587

I468	Cardiac arrest due to other underlying condition	804	0.1277	706	0.1361	98	0.0885
I213	St elevation (stemi) myocardial infarction of unsp site	786	0.1249	680	0.1311	106	0.0957
I97710	Intraoperative cardiac arrest during cardiac surgery	605	0.0961	571	0.1101	34	0.0307
I2119	Stemi involving oth coronary artery of inferior wall	587	0.0932	535	0.1031	52	0.0470
I97711	Intraoperative cardiac arrest during other surgery	541	0.0859	452	0.0871	89	0.0804
I2109	Stemi involving oth coronary artery of anterior wall	379	0.0602	333	0.0642	46	0.0415
R092	Respiratory arrest	368	0.0585	317	0.0611	51	0.0461
I219	Acute myocardial infarction, unspecified	294	0.0467	257	0.0495	37	0.0334
I2129	Stemi involving oth sites	118	0.0187	*	*	*	*
I2102	Stemi involving left anterior descending coronary artery	116	0.0184	*	*	*	*
I21A9	Other myocardial infarction type	110	0.0175	*	*	*	*
I2111	Stemi involving right coronary artery	100	0.0159	*	*	*	*
I222	Subsequent non-st elevation (nstemi) myocardial infarction	98	0.0156	*	*	*	*
I97111	Postprocedural cardiac insufficiency following other surgery	48	0.0076	*	*	*	*
I220	Subsequent stemi of anterior wall	28	0.0044	*	*	*	*
I221	Subsequent stemi of inferior wall	24	0.0038	*	*	*	*
I2121	Stemi involving left circumflex coronary artery	21	0.0033	*	*	*	*
I229	Subsequent stemi of unsp site	17	0.0027	*	*	*	*
I228	Subsequent stemi of sites	14	0.0022	14	0.0027	0	0.0000
I2101	Stemi involving left main coronary artery	*	*	*	*	*	*
T8111XS	Postprocedural cardiogenic shock, sequela	*	*	*	*	*	*
K922	Gastrointestinal hemorrhage, unspecified	6,029	0.9577	5,015	0.9667	1,014	0.9156
K56699	Other intestnl obst unsp as to partial versus complete obst	1,510	0.2399	1,354	0.2610	156	0.1409
K254	Chronic or unspecified gastric ulcer with hemorrhage	1,092	0.1735	956	0.1843	136	0.1228
K264	Chronic or unspecified duodenal ulcer with hemorrhage	850	0.1350	753	0.1452	97	0.0876
K2971	Gastritis, unspecified, with bleeding	485	0.0770	408	0.0787	77	0.0695
K251	Acute gastric ulcer with perforation	328	0.0521	267	0.0515	61	0.0551
K261	Acute duodenal ulcer with perforation	292	0.0464	243	0.0468	49	0.0442
K266	Chronic or unsp duodenal ulcer w both hemorrhage and perf	180	0.0286	155	0.0299	25	0.0226
K250	Acute gastric ulcer with hemorrhage	165	0.0262	144	0.0278	21	0.0190
K2901	Acute gastritis with bleeding	151	0.0240	125	0.0241	26	0.0235
K260	Acute duodenal ulcer with hemorrhage	134	0.0213	115	0.0222	19	0.0172
K2961	Other gastritis with bleeding	124	0.0197	101	0.0195	23	0.0208
K274	Chronic or unsp peptic ulcer, site unsp, with hemorrhage	111	0.0176	92	0.0177	19	0.0172
K256	Chronic or unsp gastric ulcer w both hemorrhage and perf	87	0.0138	69	0.0133	18	0.0163
K2981	Duodenitis with bleeding	78	0.0124	62	0.0120	16	0.0144

K284	Chronic or unspecified gastrojejunal ulcer with hemorrhage	77	0.0122	*	*	*	*
K2951	Unspecified chronic gastritis with bleeding	65	0.0103	*	*	*	*
K262	Acute duodenal ulcer with both hemorrhage and perforation	44	0.0070	*	*	*	*
K252	Acute gastric ulcer with both hemorrhage and perforation	42	0.0067	*	*	*	*
K281	Acute gastrojejunal ulcer with perforation	26	0.0041	*	*	*	*
K280	Acute gastrojejunal ulcer with hemorrhage	13	0.0021	*	*	*	*
K2941	Chronic atrophic gastritis with bleeding	*	*	*	*	*	*
K2991	Gastroduodenitis, unspecified, with bleeding	*	*	*	*	*	*
K2921	Alcoholic gastritis with bleeding	*	*	*	*	*	*
K286	Chronic or unsp gastrojejunal ulcer w both hemor and perf	*	*	*	*	*	*
K2931	Chronic superficial gastritis with bleeding	*	*	*	*	*	*
K270	Acute peptic ulcer, site unspecified, with hemorrhage	*	*	*	*	*	*
K271	Acute peptic ulcer, site unspecified, with perforation	*	*	*	*	*	*
K276	Chr or unsp peptic ulcer, site unsp, w both hemor and perf	*	*	*	*	*	*
K272	Acute peptic ulcer, site unsp, w both hemorrhage and perf	*	*	*	*	*	*
N390	Urinary tract infection, site not specified	75,202	11.9463	52,741	10.1669	22,461	20.2812
A419	Sepsis, unspecified organism	23,716	3.7674	19,748	3.8068	3,968	3.5829
T814XXA	Infection following a procedure, initial encounter	7,842	1.2458	7,056	1.3602	786	0.7097
J151	Pneumonia due to pseudomonas	1,824	0.2898	1,603	0.3090	221	0.1996
J95851	Ventilator associated pneumonia	1,503	0.2388	1,365	0.2631	138	0.1246
J150	Pneumonia due to klebsiella pneumoniae	1,441	0.2289	1,280	0.2467	161	0.1454
J159	Unspecified bacterial pneumonia	1,286	0.2043	1,040	0.2005	246	0.2221
A4151	Sepsis due to escherichia coli e. coli	1,021	0.1622	857	0.1652	164	0.1481
T8112XA	Postprocedural septic shock, initial encounter	842	0.1338	744	0.1434	98	0.0885
A4189	Other specified sepsis	772	0.1226	646	0.1245	126	0.1138
A4159	Other gram-negative sepsis	716	0.1137	625	0.1205	91	0.0822
A4181	Sepsis due to enterococcus	683	0.1085	567	0.1093	116	0.1047
A4101	Sepsis due to methicillin susceptible staphylococcus aureus	662	0.1052	602	0.1160	60	0.0542
A4102	Sepsis due to methicillin resistant staphylococcus aureus	616	0.0979	488	0.0941	128	0.1156
N10	Acute pyelonephritis	470	0.0747	375	0.0723	95	0.0858
K6819	Other retroperitoneal abscess	465	0.0739	417	0.0804	48	0.0433
A4150	Gram-negative sepsis, unspecified	459	0.0729	394	0.0760	65	0.0587
A4152	Sepsis due to pseudomonas	444	0.0705	378	0.0729	66	0.0596
J158	Pneumonia due to other specified bacteria	370	0.0588	337	0.0650	33	0.0298
J13	Pneumonia due to streptococcus pneumoniae	351	0.0558	304	0.0586	47	0.0424
K6811	Postprocedural retroperitoneal abscess	275	0.0437	261	0.0503	14	0.0126
A411	Sepsis due to other specified staphylococcus	254	0.0403	207	0.0399	47	0.0424

A414	Sepsis due to anaerobes	234	0.0372	203	0.0391	31	0.0280
J154	Pneumonia due to other streptococci	232	0.0369	205	0.0395	27	0.0244
A408	Other streptococcal sepsis	187	0.0297	168	0.0324	19	0.0172
J180	Bronchopneumonia, unspecified organism	182	0.0289	154	0.0297	28	0.0253
A4153	Sepsis due to serratia	151	0.0240	140	0.0270	11	0.0099
N151	Renal and perinephric abscess	122	0.0194	106	0.0204	16	0.0144
A401	Sepsis due to streptococcus, group b	105	0.0167	*	*	*	*
A409	Streptococcal sepsis, unspecified	91	0.0145	80	0.0154	11	0.0099
J1520	Pneumonia due to staphylococcus, unspecified	77	0.0122	*	*	*	*
A412	Sepsis due to unspecified staphylococcus	70	0.0111	58	0.0112	12	0.0108
T8141XA	Infct fol a proc, superfic incisional surgical site, init	62	0.0098	*	*	*	*
T8144XA	Sepsis following a procedure, initial encounter	57	0.0091	*	*	*	*
T8143XA	Infct fol a procedure, organ and space surgical site, init	55	0.0087	*	*	*	*
J157	Pneumonia due to mycoplasma pneumoniae	53	0.0084	42	0.0081	11	0.0099
T8149XA	Infection following a procedure, other surgical site, init	47	0.0075	*	*	*	*
J1529	Pneumonia due to other staphylococcus	38	0.0060	*	*	*	*
A403	Sepsis due to streptococcus pneumoniae	35	0.0056	*	*	*	*
A400	Sepsis due to streptococcus, group a	29	0.0046	*	*	*	*
J153	Pneumonia due to streptococcus, group b	29	0.0046	*	*	*	*
A481	Legionnaires' disease	25	0.0040	*	*	*	*
B440	Invasive pulmonary aspergillosis	21	0.0033	*	*	*	*
T8140XA	Infection following a procedure, unspecified, init	21	0.0033	*	*	*	*
A413	Sepsis due to hemophilus influenzae	17	0.0027	*	*	*	*
B250	Cytomegaloviral pneumonitis	14	0.0022	14	0.0027	0	0.0000
T8142XA	Infct fol a procedure, deep incisional surgical site, init	*	*	*	*	*	*
N340	Urethral abscess	*	*	*	*	*	*
J17	Pneumonia in diseases classified elsewhere	*	*	*	*	*	*
T8112XS	Postprocedural septic shock, sequela	*	*	*	*	*	*
A3791	Whooping cough, unspecified species with pneumonia	*	*	*	*	*	*
N413	Prostatocystitis	*	*	*	*	*	*
F05	Delirium due to known physiological condition	24,594	3.9069	15,294	2.9482	9,300	8.3974
R410	Disorientation, unspecified	15,498	2.4620	12,465	2.4029	3,033	2.7386
I97820	Postproc cerebvasc infarction following cardiac surgery	1,326	0.2106	1,240	0.2390	86	0.0777
I97821	Postprocedural cerebvasc infarction following other surgery	829	0.1317	699	0.1347	130	0.1174
G9782	Oth postproc complications and disorders of nervous sys	520	0.0826	483	0.0931	37	0.0334
I97810	Intraoperative cerebvasc infarction during cardiac surgery	78	0.0124	*	*	*	*
G9781	Other intraoperative complications of nervous system	61	0.0097	*	*	*	*

I97811	Intraoperative cerebrovascular infarction during oth surgery	50	0.0079	*	*	*	*
G972	Intracranial hypotension following ventricular shunting	*	*	*	*	*	*
I9789	Oth postproc comp and disorders of the circ sys, nec	16,574	2.6329	15,542	2.9960	1,032	0.9318
N3000	Acute cystitis without hematuria	4,896	0.7778	3,209	0.6186	1,687	1.5233
T8119XA	Other postprocedural shock, initial encounter	3,740	0.5941	3,389	0.6533	351	0.3169
I9788	Oth intraoperative complications of the circ sys, nec	1,510	0.2399	1,354	0.2610	156	0.1409
N3001	Acute cystitis with hematuria	1,131	0.1797	745	0.1436	386	0.3485
T8110XA	Postprocedural shock unspecified, initial encounter	694	0.1102	622	0.1199	72	0.0650
L7634	Postproc seroma of skin, subcu following other procedure	686	0.1090	628	0.1211	58	0.0524
T81718A	Complication of artery following a procedure, nec, init	515	0.0818	466	0.0898	49	0.0442
K91872	Postproc seroma of a dgstv sys org fol a dgstv sys procedure	292	0.0464	266	0.0513	26	0.0235
M96842	Postproc seroma of a ms structure fol a ms sys procedure	172	0.0273	151	0.0291	21	0.0190
T8172XA	Complication of vein following a procedure, nec, init	146	0.0232	*	*	*	*
T888XXA	Oth complications of surgical and medical care, nec, init	119	0.0189	104	0.0200	15	0.0135
N171	Acute kidney failure with acute cortical necrosis	104	0.0165	91	0.0175	13	0.0117
N342	Other urethritis	56	0.0089	*	*	*	*
I97648	Postproc seroma of a circ sys org fol other circ sys proc	48	0.0076	*	*	*	*
G9763	Postproc seroma of a nervous sys org fol a nervous sys proc	35	0.0056	*	*	*	*
K91873	Postproc seroma of a dgstv sys org following other procedure	34	0.0054	*	*	*	*
M96843	Postproc seroma of a ms structure following other procedure	33	0.0052	*	*	*	*
L7633	Postproc seroma of skin, subcu fol a dermatologic procedure	21	0.0033	*	*	*	*
N172	Acute kidney failure with medullary necrosis	15	0.0024	*	*	*	*
N99842	Postproc seroma of a gu sys org following a gu sys procedure	15	0.0024	*	*	*	*
T81719A	Complication of unsp artery following a procedure, nec, init	14	0.0022	*	*	*	*
I97622	Postproc seroma of a circ sys org following other procedure	11	0.0017	*	*	*	*
I97641	Postproc seroma of a circ sys org following cardiac bypass	*	*	*	*	0	0.0000
N99843	Postproc seroma of a gu sys org following other procedure	*	*	*	*	0	0.0000
J95863	Postproc seroma of a resp sys org following other procedure	*	*	*	*	0	0.0000
G9764	Postproc seroma of a nervous sys org fol other procedure	*	*	*	*	0	0.0000
I97640	Postproc seroma of a circ sys org following a cardiac cath	*	*	*	*	0	0.0000
J95862	Postproc seroma of a resp sys org fol a resp sys procedure	*	*	*	*	0	0.0000
D7833	Postprocedural seroma of the spleen fol proc on spleen	*	*	*	*	*	*
T8119XD	Other postprocedural shock, subsequent encounter	*	*	*	*	0	0.0000
T8119XS	Other postprocedural shock, sequela	*	*	*	*	0	0.0000

**N179**	**Acute kidney failure, unspecified**	127,051	20.1829	104,428	20.1307	22,623	20.4275
**N170**	**Acute kidney failure with tubular necrosis**	27,917	4.4348	24,696	4.7607	3,221	2.9084
**N990**	**Postprocedural (acute) (chronic) kidney failure**	2,273	0.3611	2,102	0.4052	171	0.1544
**N178**	**Other acute kidney failure**	659	0.1047	570	0.1099	89	0.0804
**J9601**	**Acute respiratory failure with hypoxia**	53,746	8.5379	45,612	8.7927	8,134	7.3446
**J690**	**Pneumonitis due to inhalation of food and vomit**	20,511	3.2583	15,169	2.9241	5,342	4.8236
**J95821**	**Acute postprocedural respiratory failure**	17,398	2.7638	15,124	2.9155	2,274	2.0533
**J9600**	**Acute respiratory failure, unsp w hypoxia or hypercapnia**	14,964	2.3771	12,656	2.4397	2,308	2.0840
**J9621**	**Acute and chronic respiratory failure with hypoxia**	10,656	1.6928	9,084	1.7511	1,572	1.4194
**J9602**	**Acute respiratory failure with hypercapnia**	9,144	1.4526	8,010	1.5441	1,134	1.0239
**J9611**	**Chronic respiratory failure with hypoxia**	6,353	1.0092	5,209	1.0041	1,144	1.0330
**J951**	**Acute pulmonary insufficiency following thoracic surgery**	5,308	0.8432	5,117	0.9864	191	0.1725
**J9589**	**Oth postproc complications and disorders of resp sys, nec**	4,940	0.7848	4,420	0.8520	520	0.4695
**J95811**	**Postprocedural pneumothorax**	4,872	0.7740	4,607	0.8881	265	0.2393
**J984**	**Other disorders of lung**	4,430	0.7037	4,087	0.7879	343	0.3097
**J810**	**Acute pulmonary edema**	4,400	0.6990	3,945	0.7605	455	0.4108
**J9690**	**Respiratory failure, unsp, unsp w hypoxia or hypercapnia**	4,000	0.6354	3,383	0.6521	617	0.5571
**J9622**	**Acute and chronic respiratory failure with hypercapnia**	3,996	0.6348	3,474	0.6697	522	0.4713
**J9691**	**Respiratory failure, unspecified with hypoxia**	3,514	0.5582	3,001	0.5785	513	0.4632
**J9610**	**Chronic respiratory failure, unsp w hypoxia or hypercapnia**	3,427	0.5444	2,801	0.5400	626	0.5652
**J952**	**Acute pulmonary insufficiency following nonthoracic surgery**	2,856	0.4537	2,513	0.4844	343	0.3097
**J95812**	**Postprocedural air leak**	2,370	0.3765	2,297	0.4428	73	0.0659
**J9620**	**Acute and chr resp failure, unsp w hypoxia or hypercapnia**	1,467	0.2330	1,202	0.2317	265	0.2393
**J80**	**Acute respiratory distress syndrome**	1,060	0.1684	962	0.1854	98	0.0885
**R0603**	**Acute respiratory distress**	979	0.1555	808	0.1558	171	0.1544
**J9612**	**Chronic respiratory failure with hypercapnia**	951	0.1511	780	0.1504	171	0.1544
**J95822**	**Acute and chronic postprocedural respiratory failure**	804	0.1277	688	0.1326	116	0.1047
**J9692**	**Respiratory failure, unspecified with hypercapnia**	700	0.1112	595	0.1147	105	0.0948
**J954**	**Chemical pneumonitis due to anesthesia**	119	0.0189	91	0.0175	28	0.0253
**J9588**	**Oth intraoperative complications of respiratory system, nec**	100	0.0159	*	*	*	*
**J953**	**Chronic pulmonary insufficiency following surgery**	96	0.0153	*	*	*	*
**J955**	**Postprocedural subglottic stenosis**	*	*	*	*	*	*
**J95859**	**Other complication of respirator ventilator**	*	*	*	*	0	0.0000
**T8131XA**	**Disruption of external operation (surgical) wound, nec, init**	2,872	0.4562	2,582	0.4977	290	0.2619
**T8132XA**	**Disruption of internal operation (surgical) wound, nec, init**	2,450	0.3892	2,243	0.4324	207	0.1869
**T8130XA**	**Disruption of wound, unspecified, initial encounter**	1,202	0.1909	1,067	0.2057	135	0.1219

T8133XA	Disruption of traumatic injury wound repair, init encntr	17	0.0027	*	*	*	*
T798XXA	Other early complications of trauma, initial encounter	16	0.0025	*	*	*	*
K282	Acute gastrojejunal ulcer w both hemorrhage and perforation	*	*	*	*	0	0.0000

*Values suppressed in accordance with Centers for Medicare and Medicaid Services policy

Supplementary Table 3. Adjusted and unadjusted outcomes accounting for death as a competing risk

	Unadjusted			Adjusted		
	Non-Dementia	Dementia		Non-Dementia	Dementia	
		HR (95% CI)	p-value		HR (95% CI)	p-value
**Discharge from index encounter**	REF	1.00 (0.99, 1.01)	0.55	REF	0.94 (0.93, 0.95)	<.001
**Discharge from SNF stay** [a] **(n=270378)**	REF	0.61 (0.60, 0.62)	<.001	REF	0.79 (0.78, 0.80)	<.001
90-day **Any Intensive intervention**	REF	0.90 (0.87, 0.93)	<.001	REF	1.13 (1.08, 1.18)	<.001
90-day Prolonged intubation	REF	0.85 (0.81, 0.89)	<.001	REF	1.11 (1.03, 1.20)	0.006
90-day Prolonged renal replacement therapy[b] (n=838182)	REF	0.45 (0.38, 0.53)	<.001	REF	0.59 (0.47, 0.75)	<.001
90-day ECMO	REF	0.12 (0.09, 0.27)	<.001	REF	0.49 (0.20, 1.23)	0.13
90-day Tracheostomy[c] (n=857621)	REF	0.64 (0.59, 0.69)	<.001	REF	0.99 (0.99, 1.12)	0.89
90-day Cardiopulmonary resuscitation	REF	0.57 (0.54, 0.61)	<.001	REF	0.76 (0.69, 0.83)	<.001
90-day Feeding tube[d] (n= 853217)	REF	1.41 (1.35, 1.46)	<.001	REF	1.64 (1.55, 1.74)	<.001
**Any intensive intervention during index encounter**	REF	0.69 (0.67, 0.71)	<.001	REF	0.92 (0.88, 0.96)	<.001
Prolonged intubation during index encounter	REF	0.65 (0.62, 0.69)	<.001	REF	0.96 (0.88, 1.05)	0.34
Prolonged renal replacement therapy	REF	0.31 (0.25, 0.38)	<.001	REF	0.48 (0.35, 0.66)	<.001

For Review Only

during index encounter[e] (n=838182)						
ECMO during index encounter	REF	0.13 (0.07, 0.23)	<.001	REF	0.43 (0.17, 1.12)	0.08
Tracheostomy during index encounter[f] (N=857621)	REF	0.50 (0.46, 0.54)	<.001	REF	0.84 (0.74, 0.96)	0.008
Cardiopulmonary resuscitation during index encounter	REF	0.47 (0.44, 0.50)	<.001	REF	0.66 (0.60, 0.72)	<.001
Feeding tube during index encounter[g] (n=853217)	REF	1.04 (0.99, 1.08)	0.12	REF	1.29 (1.21, 1.38)	<.001

Models were adjusted for patient-level (age, sex, race, Elixhauser index score, frailty, dual Medicare/Medicaid eligibility) and procedure-level (admission urgency, procedure risks, number of high-risk surgeries performed during the index admission)

[a] The likelihood of being discharged alive from SNF. Analysis restricted to patients who had an SNF stay within three days after discharge from index encounter and with four months of continuous Medicare FFS enrollment after discharge (or continuous enrollment till death).
[b] Patients with any ICD-10-Dx code Z99.2 during the index admission before the date of surgery or within one year before the index admission were removed from the analysis.
[c] Patients with any ICD-10-Dx code Z93.0 or Z43.0 during the index admission before the date of surgery or within one year before the index admission were removed from the analysis.
[d] Patients with any ICD-10-Dx code Z93.1 or Z43.1 during the index admission before the date of surgery or within one year before the index admission were removed from the analysis.

For Review Only
